# Magnetic Ground State Discrimination of a Polyradical Nanographene Using Nickelocene-Functionalized Tips

**DOI:** 10.1021/jacs.5c11722

**Published:** 2025-10-20

**Authors:** Diego Soler-Polo, Oleksandr Stetsovych, Manish Kumar, Benjamin Lowe, Ana Barragán, Zhiqiang Gao, Andrés Pinar Solé, Hao Zhao, Elena Pérez-Elvira, Goudappagouda, David Écija, Akimitsu Narita, Pavel Jelínek, José I. Urgel

**Affiliations:** † 86889 Institute of Physics of the Czech Academy of Science, CZ-16200 Praha, Czech Republic; ‡ Organic and Carbon Nanomaterials Unit, 508336 Okinawa Institute of Science and Technology Graduate University 1919-1 Tancha, Onna-son, Kunigami-gun, Okinawa 904-0495, Japan; § 202533 IMDEA Nanoscience, Madrid 28049, Spain; ∥ Unidad de Nanomateriales avanzados, IMDEA Nanoscience, Unidad asociada al CSIC por el ICMM, 28049 Madrid, Spain

## Abstract

Molecular magnets are a promising class of materials with exciting properties and applications. However, a profound understanding and application of such materials depend on the accurate detection of their electronic and magnetic properties. Despite the availability of experimental techniques that can sense the magnetic signal, the exact determination of the spin ground states and spatial distribution of the exchange interaction of strongly correlated single-molecule magnets remain challenging. Here, we demonstrate that scanning probe microscopy with a nickelocene-functionalized probe can distinguish between nearly degenerate multireference ground states of single-molecule π-magnets and map their spatial distribution of the exchange interaction. This method expands the already outstanding imaging capabilities of scanning probe microscopy for characterizing the chemical and electronic structures of individual molecules, paving the way for the study of strongly correlated molecular magnets with unprecedented spatial resolution.

## Graphical abstract

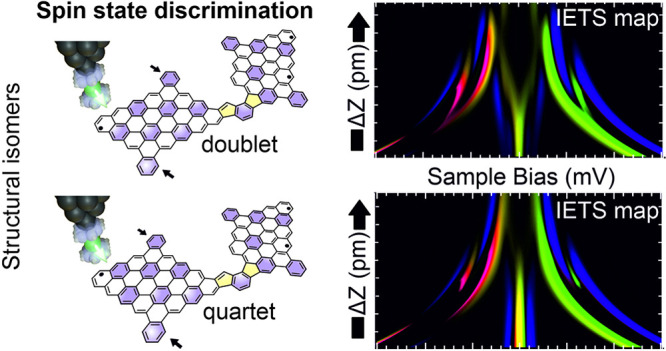

## Introduction

Single-molecule magnets represent an interesting class of materials with great application potential. One of the key factors for their future use in optoelectronics and spintronics depends on the ability to characterize their electronic and magnetic properties at the single-molecule level.^1^ Traditional molecular magnets are based on metal–organic compounds where the magnetic moment originates from strongly localized d- and f-electrons on metal centers.^2,3^ On the other hand, the magnetism of organic carbon-based radicals, the so-called π-magnetism,^4,5^ is mainly associated with highly delocalized unpaired π-electrons.^6^ Thus, a deeper understanding of the π-magnets depends not only on determining the correct magnetic ground state of a given molecule but also on our ability to resolve the spatial distribution of the inhomogeneous magnetic signal.

Traditional techniques, such as Electron Paramagnetic Resonance (EPR) and Superconducting Quantum Interference Device (SQUID) magnetometry, are extensively employed to study the magnetic properties of many organic radicals.^7,8^ While EPR provides valuable insights into the electronic environments of unpaired electrons, SQUID magnetometry is capable of measuring the total magnetic moment of bulk samples. These techniques can be complemented by Electron–nuclear double resonance spectroscopy (ENDOR),^9^ Variable Magnetic Field Scanning (VMS), and Magneto-Optical Kerr Effect (MOKE), among others, to provide a more comprehensive understanding of the magnetization and other magnetic characteristics of targeted organic radicals. All these methods, though powerful, are primarily designed for the characterization of molecular assemblies and are often limited by low sensitivity for defects or spurious intermolecular interactions. In addition, the unambiguous determination of the ground magnetic state of strongly correlated molecules with nearly degenerate electronic states represents a nontrivial task for all these methods. But more importantly, all these techniques have limited capability to provide detailed information about the spatial distribution of the magnetic signal on a single molecule. The spatial distribution is especially relevant for molecular π-magnets, where the magnetic moment is determined by strongly delocalized unpaired π-electrons, which can create a spatially inhomogeneous exchange interaction.

In recent decades, the characterization of single-molecule π-magnets with tailor-made magnetic ground states^10–12^ and the prospect for spintronics and quantum technologies^4,13–15^ has been expanded beyond traditional methods to include advanced surface science techniques. This expansion is driven by the emergent concept of on-surface synthesis,^16^ which enables the fabrication of otherwise unstable π-magnets on metal surfaces facilitated by the employed ultrahigh vacuum (UHV) environment. Moreover, this concept can be naturally complemented with low-temperature UHV scanning probe microscopy (SPM) techniques, which provide valuable insights into the chemical and electronic structure of single molecules with unprecedented spatial resolution.^6^ Nevertheless, a reliable determination of their magnetic ground and excited states remains a significant challenge.^9^ Typically, the magnetic nature of on-surface synthesized molecules with well-defined magnetic ground states, i.e., open-shell nanographenes (NGs), is inferred from the spectroscopic features acquired with SPM tip. Examples are Kondo-like resonances, Coulomb gaps, or inelastic spin-flip excitations with spatially resolved magnetic signals.^17–19^ These experimental findings are usually reinforced by state-of-the-art multireference calculations that predict the ground state of the studied nanostructures. However, this might not be enough to discriminate the magnetic state or systems with nearly degenerate states. Identifying then the nature of the ground state is hindered by similar spectral features and excitation values below the precision of ab initio multireference calculations.

Recently, EPR has been combined with Scanning Tunneling Microscopy (STM), integrating the precision of STM with the spin-sensitive capabilities of EPR.^20^ EPR-STM combines the energy resolution of EPR with the atomic-scale precision of STM.^20^ It can be used to image, characterize, and coherently control spins on surfaces. However, its application has thus far been mostly limited to metal atoms^21^ or metal-containing molecules^22^ adsorbed on MgO bilayers on Ag(100). Note that recently ESR signal was also detected by STM probe functionalized by a single molecule.^23^ In addition to the EPR-STM technique, other well-known surface-sensitive techniques like X-ray Magnetic Circular Dichroism (XMCD) and Light Magnetic Dichroism (LMD) are often used to study the magnetic properties of transition metals and heavier elements. Unfortunately, these techniques present difficulties when applied to carbon-based nanomaterials due to the low atomic number of carbon atoms and the weak magnetic response, together with the need for well-defined surface structures. Another alternative is spin-polarized STM with sharp magnetic tips,^24^ which employs the effect of tunnel magnetoresistance to achieve atomic-scale spin contrast. Nevertheless, this method cannot discriminate between different molecular spin states. Also, the significant chemical reactivity of atomically sharp magnetic tips hampers stable scanning conditions at close tip–sample distances.

Under this scenario, tip functionalization with a nickelocene (NiCp_2_) molecule as spin-sensitive probes or magnetic field sensors (with or without the presence of a magnetic field)^25–31^ has recently emerged as an innovative approach providing quantitative spin-dependent information.^28^ The NiCp_2_ molecule has total spin *S* = 1, where the spin–orbit coupling causes a split of the triplet state into the in-plane spin ground state (*m*_s_ = 0) and the doubly degenerate spin-up and spin-down excited state (*m*_s_= ± 1). Importantly, the net spin (*S* = 1) of NiCp_2_ placed on the metallic tip apex remains preserved from scattering events with itinerant electrons from the metal tip. Thus, inelastic electron tunneling spectroscopy (IETS) acquired with a NiCp_2_-functionalized probe shows a large enhancement of the inelastic signal at 4 meV as a consequence of the spin excitation from the ground to the first excited state.^25^ The presence of the exchange interaction between a NiCp_2_-probe and a magnetic system on the surface can modify the characteristic IETS signal along SPM tip approach, as shown in the inset of Figure 1. Therefore, the variation of the IETS signal at short tip–sample distances provides valuable information about the exchange interaction as well as the spin states of the inspected system by NiCp_2_-probe. This strategy was employed to probe the magnetic signal of single atoms,^25^ molecules,^26^ one-dimensional metal–organic chains,^29^ and 2D materials.^32^ In this work, we demonstrate the potential for NiCp_2_-functionalized tips as spin-sensitive probes toward the precise discrimination of magnetic ground states of strongly correlated polyradical molecules, where other aforementioned methods often struggle to resolve subtle differences between spin configurations. It also enables us to map the inhomogeneous spatial distribution of the local exchange interaction of the polyradical π-magnet acting on an external spin. We show these on three different polyradical nanographenes: diradical **D1** (see Figure 2a) and two isomeric triradicals **D2a**, **D2b** (see Figures 3a,g). Using scanning tunneling spectroscopy (STS) and IETS acquired with NiCp_2_-functionalized tips at a temperature of ∼4.4 K, and supported by theoretical analysis, we can not only distinguish different spin multiplets but also resolve the number of unpaired spins, demonstrating a distinct response between the diradical **D1** and the triradicals **D2a**, **D2b**. More importantly, this method allows us to resolve the nearly degenerate ground states of the triradical dimers **D2**, confirming for **D2a** and **D2b**, respectively, the triradical doublet and quartet as their ground states. This discrimination highlights the ability of NiCp_2_-functionalized tips to resolve the spatial distribution of spins and offers a clear advantage for spin mapping at the molecular level. The experimental findings are complemented by many-body calculations employing the complete active space configuration interaction (CASCI) method.

**Figure 1:**
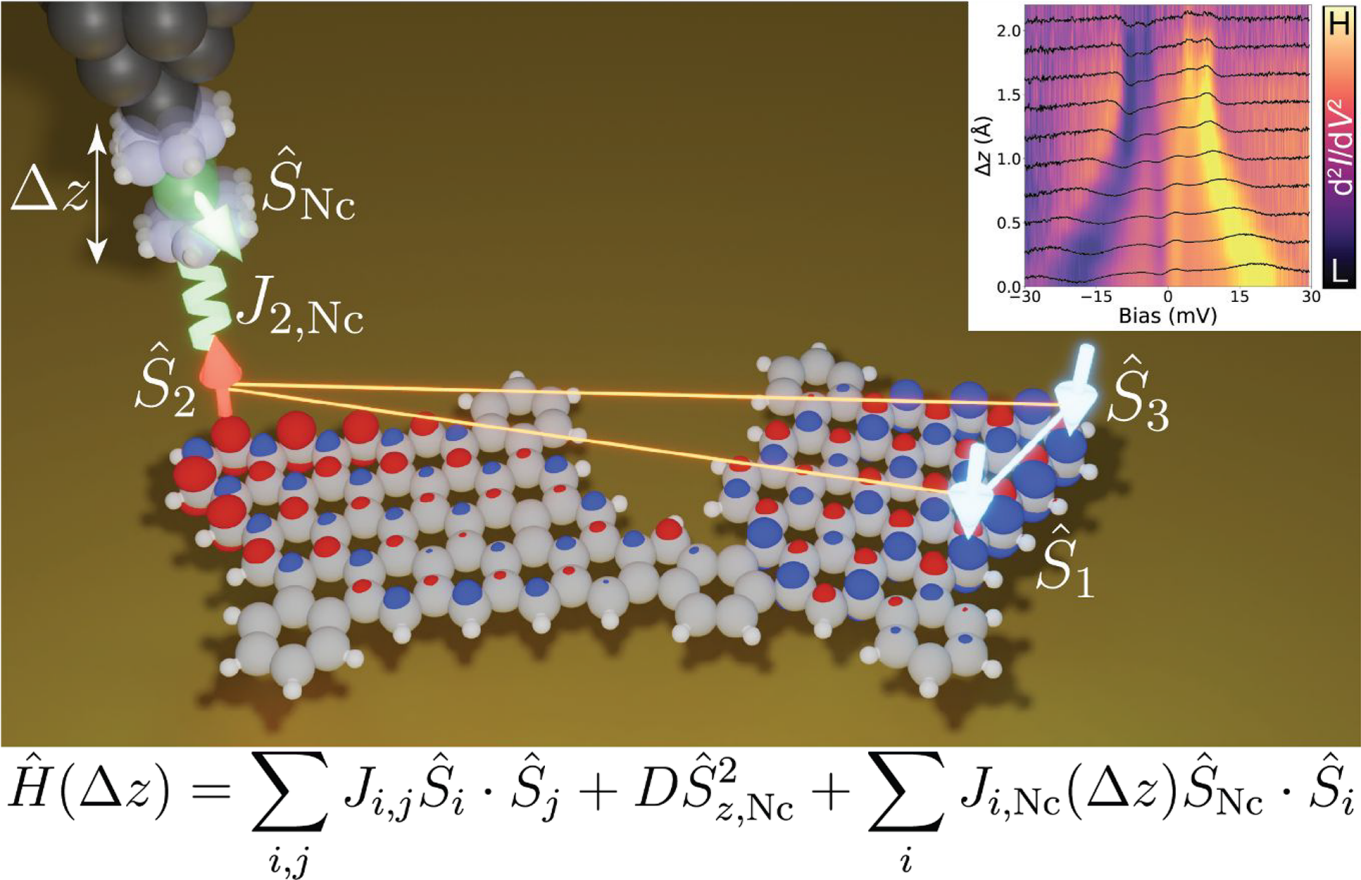
Schematic representation of the spin model. A polyradical molecule is fitted to a spin model which is further coupled to the *S* = 1 site modeling the NiCp_2_ tip, illustrated schematically by the four arrows. Color scale: gray—C, white—H, green—Ni, black—metallic atoms of the STM tip. Blue and red lobes represent the spin density of the molecule calculated by multireference CASCI method. Inset: tip height (Δ*z*) dependent d^2^ *I*/d*V* ^2^ spectra, experimentally acquired using a NiCp_2_-functionalized tip, that are characteristic of the molecule’s magnetic ground state.

**Figure 2:**
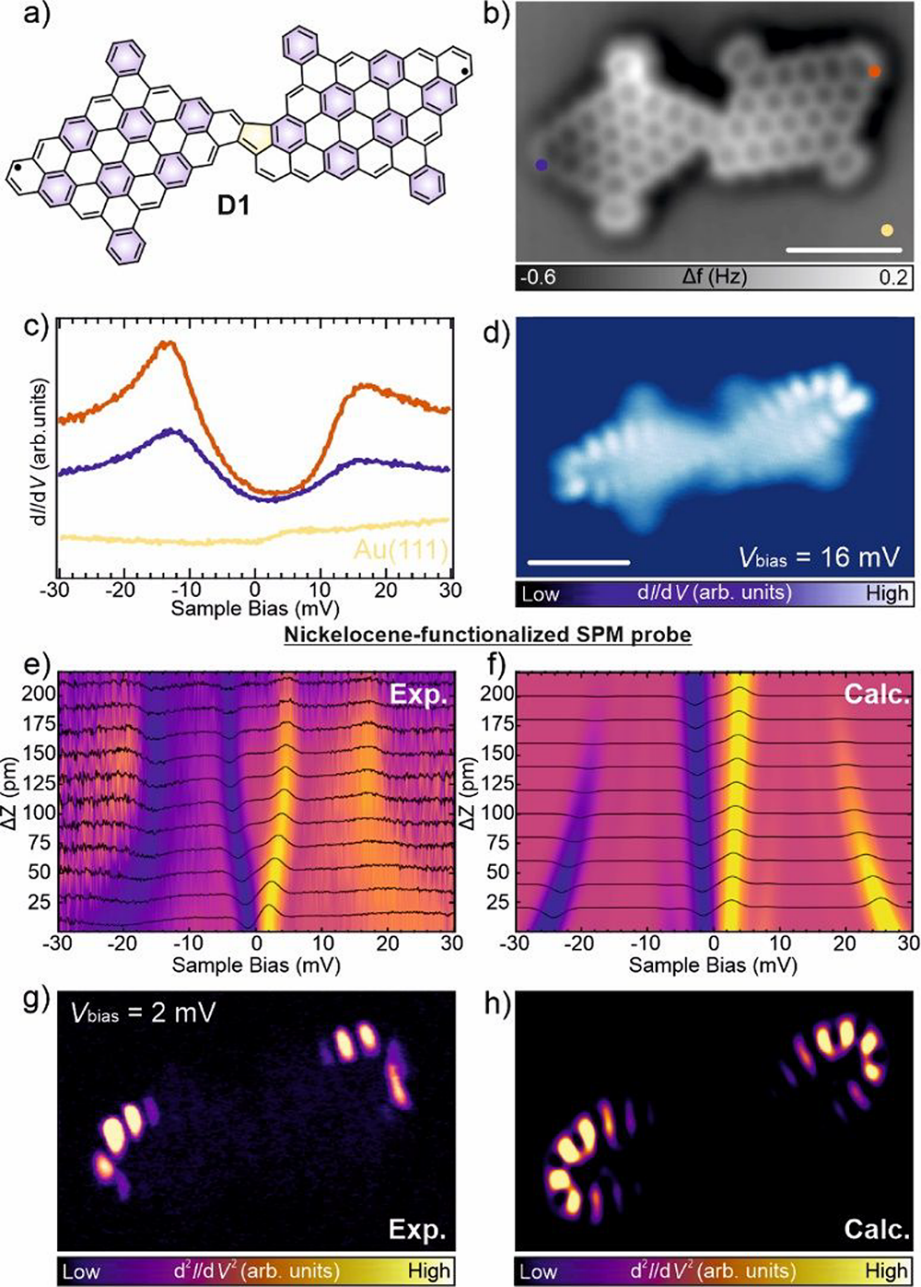
Structural and electronic characterization of D1 on Au(111). (a) Chemical sketch of dimer D1 composed of two I NGs linked through a pentagon following an oxidative ring closure reaction. (b) Experimental constant-height nc-AFM image acquired with a CO-tip confirming the structure of D1. Scale bar = 1 nm. (c) d*I*/d*V* spectra of D1 acquired with a CO-tip at the positions marked with orange and blue circles in (a). From both spectra, we infer a *J*_eff_ ∼ 12 meV. The yellow circle corresponds to the reference spectrum acquired on the bare Au(111) substrate. Spectra are normalized and offset for clarity. Set points: *V*_b_ = 30 mV, *I*_t_ = 45 pA (orange, blue), *V*_b_ = 30 mV, *I*_t_ = 100 pA (yellow). (d) Constant-height d*I*/d*V* map of D1 acquired with a CO-tip at *V*_b_ = 16 mV. Scale bar = 1 nm. (e) Height-dependent map composed of a series of 11 d^2^ *I*/d*V* ^2^ spectra acquired with a NiCp_2_ terminated scanning-probe tip on D1 edges (f) Theoretical simulation of the d^2^ *I*/d*V* ^2^ spectra corresponding to the experiment shown in (e). (g) Constant-height d^2^ *I*/d*V* ^2^ map of D1 acquired with a NiCp_2_-functionalized tip at *V*_b_ = 2 mV. (h) Simulated constant-height d^2^ *I*/d*V* ^2^ map obtained by computing the overlap of NiCp_2_ orbitals with the molecular spin density.

**Figure 3:**
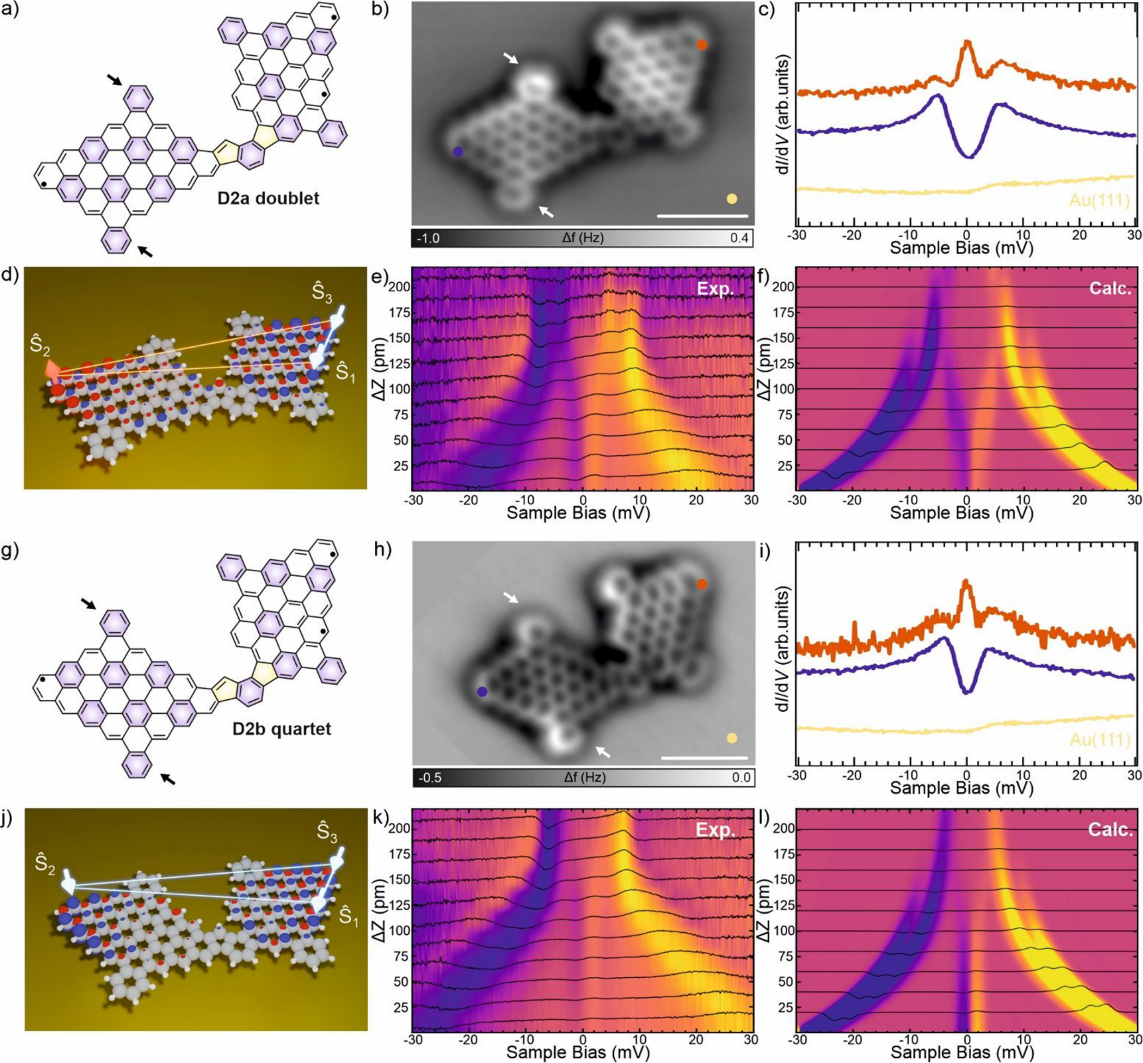
Structural and electronic characterization of dimers **D2** on Au(111). (a, g) Chemical sketches of dimers **D2a** and **D2b**. (b, h) Constant-height nc-AFM images acquired with a CO-tip confirm the structure of dimers **D2a** and **D2b**. Scale bars = 1 nm. (c-i) STS measurements acquired with a CO-tip at the positions indicated in (b and h). Spectra are normalized and offset for clarity. Set points: (c) *V*_b_ = 30 mV, *I*_*t*_ = 110 pA (purple), *I*_*t*_ = 60 pA (orange), *I*_*t*_ = 100 pA (gold). (i) *V*_b_ = 30 mV, *I*_*t*_ = 60 pA (purple), *I*_*t*_ = 30 pA (orange), *I*_*t*_ = 100 pA (gold). (d, j) Schematic spin models with doublet and quartet ground states employed to model Dimer **D2a** and **D2b**, respectively. In both cases spins *Ŝ*_1_ and *Ŝ*_3_ are ferromagnetically coupled. *Ŝ*_2_ is antiferromagnetically coupled to *Ŝ*_1_ and *Ŝ*_3_ in the case of the doublet (d) and ferromagnetically coupled to *Ŝ*_1_ and *Ŝ*_3_ in the case of the quartet (j). Blue/red lobes indicate the spin density calculated by the CASCI method. (e, k) Tip-height dependent maps composed of a series of 11 d^2^ *I*/d*V* ^2^ spectra acquired with a NiCp_2_ terminated scanning-probe tip on the left wings (purple markers in b, h) of **D2a** and **D2b** at different tip–sample distances (20 pm between spectra). (f, l) Theoretical simulations of the d^2^ *I*/d*V* ^2^ spectra corresponding to the experiments shown in (e and k), obtained for the doublet and quartet ground state sketched in (d and j), respectively.

## Results and Discussion

To rationalize the variation of the experimental IETS signal, we combine Heisenberg spin models and the cotunneling theory^33^ to simulate the corresponding inelastic tunneling current.^28^ The Heisenberg model describes the tip–sample height-dependent exchange interaction *J*_Nc,*i*_(*z*) between a set of molecular 1/2-spins *Ŝ*_*i*_ and spin *Ŝ*_Nc_ = 1 of the NiCp_2_ tip, schematically shown in Figure 1. The spin Hamiltonian *Ĥ*_Nc_ of NiCp_2_ tip is given by the out-of-plane anisotropy *Ĥ*_Nc_ = *DŜ*_Nc,*z*_^2^, with *D* = 4 meV. The spin Hamiltonian of the molecule is described by a set of spin 1/2 sites: H^M=∑i,jJi,jS^i·S^j , where the coefficients *J*_*i*,*j*_ are chosen to reproduce the energy spectrum of the molecule obtained from many-body CAS calculations. Lastly, we include the tip–sample dependent exchange interaction *J*_Nc,*i*_ between the local molecular spins *Ŝ*_*i*_ and the NiCp_2_ *Ŝ*_Nc_, H^int⁡(z)=∑iJNc,i(Δz)S^Nc·S^i . Here, Δ*z* represents the variation of the tip–sample distance with respect to the closest distance. The exchange interaction between the NiCp_2_ spin and local spins is defined as *J*_Nc,*i*_(Δ*z*) = *J*_0,*i*_ exp­(−λΔ*z*). We can now write the full Heisenberg spin model as

H^M,Nc(Δz)=H^M+H^Nc+H^int⁡(Δz)=∑i,jJi,jS^i·S^j+DS^Nc,z2+∑iJNc,i(Δz)S^Nc·S^i 1

However, the eigenvalues of the Heisenberg spin model ([Disp-formula eq1]) cannot be compared directly to the experimental IETS signal, as the intensity of IETS peaks corresponding to given spin excitations may strongly alternate. To account for the relative intensity of the different eigenstates of the spin model, we resort to the cotunneling theory to calculate the inelastic current through the spin system ([Disp-formula eq1]) coupled to reservoirs.^28^

For the experimental part, we followed an on-surface synthesis approach to fabricate the three polyradical NGs (a diradical, **D1,** and the two isomeric triradicals **D2a**, **D2b**) by depositing a suitable molecular precursor on the Au(111) surface under UHV conditions. Subsequent annealing at 250 °C induces the oxidative ring closure and dehydrogenation reactions, together with an intermolecular coupling, which provides various nanographene products (for more details, see Figure S20).^34^ We elucidated their chemical structure by means of high-resolution noncontact atomic force microscopy (nc-AFM) with a carbon monoxide (CO)-functionalized tip (see Figure 2b and Figure 3b,h).^35^ Ab initio CASCI calculations predict that **D1** is a diradical with a singlet open-shell ground state and an excited triplet state at 12 meV. The calculations assign **D2a**, **D2b** as triradicals with nearly degenerate ground states, with the quartet and doublet states being separated by 4 meV. Such a small difference in energy makes it impossible to reliably assign the ground state using CASCI.

Next, we performed STS measurements of **D1** (see Figure 2a,b) to analyze its electronic structure. An indirect indication of the magnetic ground state of **D1** is manifested in the d*I*/d*V* spectra acquired in the vicinity of the Fermi level. Figure 2c shows such a d*I*/d*V* spectrum featuring two conductance steps at 12 meV symmetrically positioned around the Fermi energy, which we tentatively assign to inelastic spin-flip excitations from the ground state to the first excited magnetic states with excitation energy of *J*_eff_ = 12 meV. This value matches well to the calculated energy gap between the singlet ground state and the first excited triplet state by CASCI­(12,12) calculations, which provides a singlet–triplet gap of 12 meV (see Figures S1 and S2 in the SOM for a description of the employed active space and the predicted diradical character). In addition, the constant-current d*I*/d*V* maps obtained close to the spin excitation thresholds are shown in Figure 2d. They match well with the calculated d*I*/d*V* maps of Natural Transition Orbitals (NTO)^36^ corresponding to the singlet–triplet transition obtained from CASCI calculations (see Figure S3). While there is good agreement between the experimental and theoretical d*I*/d*V* maps and the inelastic energy gap (see Figures S8 and S9 for the simulated map of the spin excitation), direct experimental evidence of the presence of the singlet ground state and first excited triplet state is missing.

Figure 2e shows the *z*-dependent d^2^ *I*/d*V* ^2^ spectra acquired with NiCp_2_-probe at the edge of the **D1** (at the orange marker in Figure 2b), which reveals a significant variation of the d^2^ *I*/d*V* ^2^ signal upon NiCp_2_-probe approach. In far distance, we observe two characteristic d^2^ *I*/d*V* ^2^ peaks at 4 and 16 meV at each polarity. These two peaks correspond, respectively, to the bare excitation of the NiCp_2_ and the joint excitation of the molecule and the NiCp_2_ (12 + 4 = 16 meV). The bare excitation of the molecule, with the NiCp_2_ in its ground state, does not contribute to the current, as reproduced in the simulated map in Figure 2f (see SOM for a detailed discussion). As the NiCp_2_-probe approaches, we observe a gradual inward renormalization of the peak at 4 meV toward lower values, while the peak at 16 meV becomes broader and shifts to higher energies.

To rationalize the experimental d^2^ *I*/d*V* ^2^ spectra, we carried IETS simulations using the aforementioned theoretical model combining transport cotunneling theory with a Heisenberg model. Here, the molecule (*Ĥ*_M_ in eq 1 above) is represented by two-site two spins *Ŝ*_*i*_ = 1/2 model *Ĥ*_M_ = *JŜ*_1_·*Ŝ*_2_ where we set the parameter *J*_1,2_ = 12 meV to reproduce the singlet ground state and the singlet–triplet energy gap 12 meV obtained from CAS calculations.

Figure 2f shows the simulated *z*-dependent d^2^ *I*/d*V* ^2^ spectra that reproduce the experimental data set well. As the tip approaches, the exchange interaction increases and thus the singlet and triplet molecular states become mixed. Therefore, the spin of the molecule cannot be used as a quantum number, since its expected value deviates largely from the eigenvalues 0 and 1. This mixing of spin states at close tip–sample distances provides the characteristic renormalization corresponding to the particular singlet–triplet spin configuration of the molecule (see Figures S14 and S15 and accompanying text for a discussion of the eigenstates of the Heisenberg model and their associated quantum numbers). It is worth noting that such a renormalization is characteristic of the antiferromagnetic dimer (being absent in the case of ferromagnetic coupling, see Figure S12 and underlying discussion in SOM) and is also due to the asymmetric coupling of the spin of the NiCp_2_-probe to one of the spins *Ŝ*_*i*_ of the molecular dimer: that is, in eq 1) we set as *J*_Nc,1_(Δ*z*) = *J*_0,1_ exp (− λΔ*z*) and *J*_Nc,2_(Δ*z*) = 0. We set *J*_0,1_ = 8 meV and λ = 1.5 to fit the observed branching in the experimental range of 2 Å. These parameters are employed for all cases and the spectroscopic features later analyzed are robust to perturbation (see Figure S19) In this way, we account for the local nature of the spin interaction. If the spin of NiCp_2_-probe coupled to the whole spin of the molecule, the inward renormalization of the bare excitation of NiCp_2_ at 4 meV would not be observed (see Figure S15 and the accompanying text in the SOM for details). Also the variation of d^2^ *I*/d*V* ^2^(*z*) signal is strongly site dependent. For example in the center of the molecule, the d^2^ *I*/d*V* ^2^(*z*) signal remains constant upon the NiCp_2_-probe approach. These observations indicate a strongly inhomogeneous distribution of magnetic signal over the molecule, due to the presence of spatially extended unpaired π-electrons.

Thus, we recorded spatial IETS maps at 2 meV to capture the spatial distribution of the variation of the bare NiCp_2_ excitation. Figure 2g reveals a localization of the IETS signal predominantly at the two edges, which nicely coincides with the calculated spin-density distribution shown in Figure S2. To rationalize the experimental map, we calculated spatial NiCp_2_ maps for dimer **D1** using a Heisenberg Hamiltonian that incorporates spatially dependent exchange interactions between the NiCp_2_ tip and the molecular spin centers. The spatial exchange interactions are derived from the wave function overlap between the NiCp_2_ and molecular spin density, following Chen’s derivative rule.^37^ Using this Hamiltonian, the spatially resolved inelastic current is calculated as a function of the bias voltage, where the NiCp_2_ excitation undergoes renormalization. The very good match between the experimental and calculated NiCp_2_ spatial maps, compare Figure 2g,h, demonstrate that the NiCp_2_ spatial d*I*/d*V* maps provide an accurate representation of the molecule’s spin-density distribution, in good agreement with the radical character predicted from an ab initio analysis (see the Natural Orbitals in Figure S2 in the SOM).

Next, we focus on the electronic and magnetic properties of dimers **D2a** and **D2b**. These two dimers are structural isomers that differ only by a reflection on one-half of the molecule (see Figures 3a,g). Experimental d*I*/d*V* spectra show, in both cases, the coexistence of a Kondo peak on the right wing of the dimers and a spin excitation on the left wing (see Figures 3c,i). Ab initio CASCI­(11,11) with NEVPT2 corrections yield for the two isomers identical electronic properties: Both dimers **D2** are predicted to be fully triradical molecules with a doublet ground state and a quartet ground state at *J*_eff_ = 4 meV, with the next excited state (another triradical doublet) around 0.5 eV higher in energy (see Figures S4 and S5 in the SOM for the orbitals forming the active space). However, due to the small spectral gap between the ground and the first excited state, the theoretical calculations do not allow for a conclusive determination of the ground state. Thus, we tried to discern the ground state using the spatial distribution of low-energy d*I*/d*V* maps corresponding to spin-excitation and Kondo resonance. To rationalize the experimental STS maps shown in Figure S21, we calculated NTO and Kondo orbitals (KO) for both doublet and quartet ground states,^38^ as described in detail in SOM. The resulting theoretical d*I*/d*V* maps of NTO and KO (see Figures S8–S13) for the doublet and quartet ground states are very similar, and they match the experimental evidence very well. Therefore, the spatial distributions of the spin excitation and Kondo signal are also insufficient to determine whether the triradical ground states of **D2a** and **D2b** are doublets or quartets.

We then focus on the d^2^ *I*/d*V* ^2^ spectra acquired with NiCp_2_-probe on the left wing of the dimers **D2**, where the spin excitation is visible. As shown in Figures[Fig fig3]e,k, both **D2a** and **D2b** have curved branches which shift toward higher energies with decreasing tip–sample distance (Δ*z*). Closer to the zero bias, however, the two structural isomers exhibit different behavior. Spectra acquired for **D2a** display branches originating from the renormalized excitation lines of the NiCp_2_ excitation that progressively shift toward zero bias with decreasing Δ*z*, while spectra acquired for **D2b** show two parallel line features with unchanging energy for small and intermediate Δ*z* that disappear for large Δ*z*. Note that the STS in Figure 3c,i, acquired with a CO tip, provides the value of the spin excitations for the two systems. However, they cannot be compared directly to the maps in Figure 3e,k, since they are obtained with the magnetically functionalized tip that has the low-lying excitation of the NiCp_2_. Consequently, the branches of the maps correspond to states where the molecular spin is no longer a good quantum number. We will show that the subtle difference between Figure 3e,k is a robust indication of distinct ground states, a triradical doublet and a triradical quartet, respectively. Natural Orbitals from CASCI calculations show the presence of one radical on the left wing (where the spin excitation is visible) and two radicals on the right wing (where the Kondo signal is visible). See Figures S6 and S7 in the SOM for details. The location of these three radicals is schematically shown in Figures 3d,j. To model these spectra, we resort to a Heisenberg spin model following eq 1 with three spin sites (see Figures S16 and S17 for the local basis of orbitals on which the spin model is defined).

In this case, however, we construct three-sites molecular spin Hamiltonians, *Ĥ*_M_, one that has the doublet ground state, *Ĥ*_M,d_, and one that has the quartet ground state, *Ĥ*_M,__q_ (see Figure 3d,j). These Hamiltonians are respectively given by *Ĥ*_M,d_ = *J*_d_ *Ŝ*_1_·*Ŝ*_2_ + *J*_d_ *Ŝ*_2_·*Ŝ*_3_ + *J*_*t*_ *Ŝ*_1_·*Ŝ*_3_ and *Ĥ*_M,*q*_ = *J*_*q*_ *Ŝ*_1_·*Ŝ*_2_ + *J*_*q*_ *Ŝ*_2_·*Ŝ*_3_ + *J*_*t*_ *Ŝ*_1_·*Ŝ*_3_. The coefficients are fitted to reproduce the experimentally obtained spin excitation for, respectively, **D2a** and **D2b** (see purple curves in Figure 3c,i). This is achieved by setting *J*_d_ = 4 meV, *J*_*q*_ = −2 meV. Furthermore, we set *J*_*t*_ = −200 meV to reproduce the spin correlations and the excited doublet state of the ab initio CAS calculations (see Tables S1 and S2 in the SOM). The large ferromagnetic interaction on the right-side radicals is akin to the ferromagnetic coupling of *S* = 1 triangulenes^39^ and is consistent with the energy of the excited doublet in the Ab initio CASCI­(11,11) calculations. Therefore, Hamiltonian *Ĥ*_M,d_ has a doublet ground state and a quartet excited state at 4 meV, while Hamiltonian *Ĥ*_M,*q*_ has a quartet ground state and an excited doublet state at 2 meV. We now plug these Hamiltonians in eq 1 with *J*_Nc,2_(*z*) = *J*_0,2_ exp (−λΔ*z*) and *J*_Nc,1_(*z*) = *J*_Nc,3_(*z*) = 0 to account for the local coupling of the NiCp_2_-probe with the single radical on the left wing of the dimers **D2** (see Figures 3d,j). The resulting simulated d^2^ *I*/d*V* ^2^ spectra for the doublet and quartet model (Figures 3f,l) match well with the experimentally maps shown in Figures 3e,k including the distinct behavior of the branches close to zero bias (see Figure S23 for the raw curves employed to construct these maps). This nice agreement allows us to conclude that the dimers **D2a** and **D2b** have doublet and quartet ground states, respectively. It is important to highlight that the spectroscopic characteristics of the maps associated respectively with the doublet and quartet states remain robust despite perturbations of the spin excitation: the behavior of the central branches remains unaltered for a range of different spin excitations (see Figure S18 in SOM). This is crucial to conclude that the NiCp_2_ measurements allow us to discriminate unambiguously between different ground states of a single molecule.

## Conclusions

In conclusion, the particular spin state of a single molecular magnet, due to the exchange interaction with the nickelocene functionalized probe, generates a unique IETS signal response. This tip-height dependent IETS signal characteristic for a particular spin state allows for the discrimination of magnetic ground states of a given molecular magnet. This method significantly expands the possibilities of the SPM technique for characterizing the magnetic properties of individual molecules. At the same time, it opens new possibilities for the study of complex spin systems such as π-d or spin-frustrated molecular systems with high spatial resolution. In principle, this method can also be used for 3D mapping of the exchange field of molecular magnets. We anticipate that this method can be extended by incorporating higher-order scattering terms, enhancing its sensitivity toward complex molecular spin states.

## Supplementary Material



## Notes

Experimental details, synthesis, characterizations, and computational studies (PDF)
