# Supplementary material for: Magnetic Ground State Discrimination of a Polyradical Nanographene Using Nickelocene-Functionalized Tips
Source: J Am Chem Soc. 2025 Oct 20;147(43):39072–9. doi: 10.1021/jacs.5c11722 (PMC12576821; doi:10.1021/jacs.5c11722)
Supplement: Supplementary file 1 [file ja5c11722_si_001.pdf]

Supporting Information: Supplementary Material for: Magnetic ground state discrimination of a Polyradical Nanographene using Nickelocene-Functionalized Tips

Diego Soler-Polo,[*,†] Oleksander Stetsovych,[†] Manish Kumar,[†,‡] Benjamin Lowe,[†] Ana Barragán,[¶] Zhiqiang Gao,[§] Andrés Pinar Solé,[‖] Hao Zhao,[§] Goudappagouda,[§] David Écija,[¶,⊥] Akimitsu Narita,[*,§] Pavel Jelínek,[*,†,#] and José I. Urgel[*,¶,⊥]

†*Institute of Physics, Czech Academy of Sciences, Prague 16200, Czech Republic*

‡*Department of Condensed Matter Physics, Faculty of Mathematics and Physics, Charles University, CZ12116 Prague 2, Czech Republic*

¶*IMDEA Nanoscience, C/Faraday 9, Campus de Cantoblanco, 28049 Madrid, Spain*

§*Organic and Carbon Nanomaterials Unit, Okinawa Institute of Science and Technology Graduate University, 1919-1 Tancha, Onna-son, Kunigami-gun, Okinawa 904-0495, Japan*

‖*Center for Quantum Nanoscience (QNS)*

⊥*Unidad de Nanomateriales avanzados, Imdea Nanoscience, Unidad asociada al CSIC por el ICMM, 28049 Madrid, Spain*

#*Czech Advanced Technology and Research Institute (CATRIN), Palacký University Olomouc, 779 00 Olomouc, Czech Republic*

E-mail: soler@fzu.cz; akimitsu.narita@oist.jp; jelinekp@fzu.cz; jose-ignacio.urgel@imdea.org

Contents

1 Methods S-2

2 Simulations S-7

3 Experimental details S-17

References S-21

1 Methods

CASCI calculations

First, the geometry of free standing molecules is optimized using the DFT code FHI-AIMS code[S1] with the PBE0 functional.[S2] As the molecules have polyradical character, we have employed the many-body CASCI method for a better description of their electronic structure,[S3] resolving full many-body Hamiltonian:

$$\hat{H}_{\text{CAS}} = \sum_{i,j,\sigma} t_{ij}\hat{C}^{\dagger}_{i\sigma}\hat{C}_{j\sigma} + \sum_{i,j,k,l,\sigma,\sigma'} V_{ijkl}\hat{C}^{\dagger}_{i\sigma}\hat{C}^{\dagger}_{j\sigma'}\hat{C}_{k\sigma'}\hat{C}_{l\sigma}$$

Where $\hat{C}^{\dagger}_{i\sigma}(\hat{C}_{j\sigma})$ are the creation (annihilation) operators are associated with the basis of molecular orbitals. We have calculated the one and two-body integrals t_{ij} and V_{ijkl} from the quantum chemistry software ORCA.[S4]

For further correction of the out-of-CAS dynamical electron correlation, we employed the Quasidegenerate Second-Order N-Electron Valence State Perturbation Theory (QD-NEVPT2).[S5]

Natural Orbitals

We construct the spinless 1-particle reduced density matrix from the wavefunction obtained from our CASCI calculation.[S6] The diagonalization of the density matrix provides

the natural orbitals, and their occupations yield the number of unpaired electrons.

Natural Transition Orbitals

To rationalize the dI/dV maps of the spin excitation, we employ the so-called Natural Transition Orbitals (NTO)[S7] for the spin-flip operator encoding the transition between two different many-body spin states. NTO orbitals are obtained from the diagonalization of the matrix $TT^\dagger$, where the matrix T is given by elements

$$T_{jk} = \langle \Psi_1 | \hat{C}^\dagger_{j\uparrow} \hat{C}_{k\downarrow} | \Psi_0 \rangle,$$

where the indices j, k run over the active orbitals.

Kondo Orbitals

To account for the spatial distribution of the Kondo signals, we employ the recently developed formalism of Kondo orbitals.[S8] Here, we diagonalize the coupling matrix obtained from a multi-level Anderson Hamiltonian, which models the coupling of our molecule (described by the CASCI Hamiltonian) to the metallic substrate. This allows us to identify the antiferromagnetic channels contributing to the scattering process, which accounts for the Kondo peaks.

Simulation of dI/dV maps Theoretical dI/dV maps were calculated by the Probe Particle Scanning Probe Microscopy (PP-SPM) code[S9] for a CO-like tip, which was represented by a linear combination of PxPy (85%) and s-like (15%) orbitals without tip relaxation.

Spin models

We fit dimer **D1** to a two-site spin model given by:

$$\hat{H}_{D1} = J_1 \hat{\vec{S}}_1 \cdot \hat{\vec{S}}_2 \tag{1}$$

with $J_1 = 12$ meV. Dimers **D2** are fitted to a three-site spin model, where site 2 corresponds to the lone radical on the left-wing (see Figs. SS16b,SS17b) and sites 1 and 3 correspond to the two radicals on the right-wing of the molecule (see Figs. SS16b,c,SS17b,c). The fitting can be done to reproduce either a doublet or a quartet ground state:

$$\hat{H}_{D2,d} = J_t \vec{\hat{S}}_1 \cdot \vec{\hat{S}}_3 + J_d \left(\vec{\hat{S}}_1 + \vec{\hat{S}}_3 \right) \cdot \vec{\hat{S}}_2 \tag{2}$$

$$\hat{H}_{D2,q} = J_t \vec{\hat{S}}_1 \cdot \vec{\hat{S}}_3 + J_q \left(\vec{\hat{S}}_1 + \vec{\hat{S}}_3 \right) \cdot \vec{\hat{S}}_2, \tag{3}$$

with the coefficients for the spin models as specified in the main text.

Calling in general $\hat{H}_M$ to any of the molecular spin hamiltonians $\hat{H}_{D1}, \hat{H}_{D2,d}$ or $\hat{H}_{D2,q}$ above, our goa is now to connect such hamiltonian to the NiCp2 tip. This tip is described by a S=1 site with a out-of-plane magnetic anisotropy of 4 meV, $\hat{H}_{Nc} = D\hat{S}^2_{Nc,z}$.

To complete the model, we need to specify how the Nickelocene tip coupled to each nanographene through the interaction hamiltonian $\hat{H}_{\text{int}}(z)$, where z is the tip-sample distance. The S=1 site is coupled via a magnetic anti-ferromagnetic interaction that arises from the kinetic exchange. In its greatest generality, this interaction Hamiltonian has then the form

$$\hat{H}_{\text{int}}(z) = \sum_i J_{\text{Nc},i}(z) \hat{S}_{Nc} \cdot \hat{S}_i, \tag{4}$$

where the couplings $J_{\text{Nc},i}(z)$ will decay exponentially with height, $J_{\text{Nc},i}(z) = J_{0,i} \exp(-\lambda z)$. However, due to the localization of the radicals on the nano-graphenes (see Fig. SS2 and Fig. SS6), the spin of the Nickelocene couples only to one site of the model We in general have a spin Hamiltonian:

$$\hat{H}_{M,\text{Nc}}(z) = \hat{H}_M + \hat{H}_{\text{Nc}} + \hat{H}_{\text{int}}(z). \tag{5}$$

Cotunneling Theory To calculate the d^2I/dV^2 maps from the spin models, we employ

the perturbative approach described in reference[S10] , as implemented for the case of NiCp2 tips and molecular systems.[S11] The cotunneling results from a second-order perturbation calculation starting from the Hamiltonian

$$\hat{H}(z) = \hat{H}_{M,\mathrm{Nc}}(z) + \hat{H}_{\mathrm{tip}} + \hat{H}_{\mathrm{sub}} + \hat{H}_{\mathrm{tun}}(z), \tag{6}$$

where $\hat{H}_{M,\mathrm{Nc}}$ is given by eq. 5, $\hat{H}_\eta = \sum_{k,\sigma} \varepsilon_{\eta,k,\sigma}, \hat{a}^\dagger_{\eta,k,\sigma}\hat{a}_{\eta,k,\sigma}$, with $\eta = \mathrm{tip}, \mathrm{sub}$, and $\hat{H}_{\mathrm{tun}}$ is the tunneling hamiltonian[S12] given by

$$\begin{aligned}\hat{H}_{\mathrm{tun}}(z) = \sum_{i,\sigma,\sigma'} T_{0,k,k'} \left(\hat{a}^\dagger_{\mathrm{tip},k,\sigma}\hat{a}_{\mathrm{sub},k,\sigma'} + \mathrm{h.c.}\right) + \\ \sum_{i,\sigma,\sigma'} T_{i,k,k'}(z)\vec{P}_{\sigma,\sigma'} \cdot \vec{\hat{S}}_i \left(\hat{a}^\dagger_{\mathrm{tip},k,\sigma}\hat{a}_{\mathrm{sub},k,\sigma'} + \mathrm{h.c.}\right),\end{aligned} \tag{7}$$

where i labels the sites of the spin Hamiltonian $\hat{H}_{M,\mathrm{Nc}}$, $\vec{P}$ are the $\frac{1}{2}$ Pauli matrices and $T_{i,k,k'}(z)$ are the coupling between electrons and spins, which include a dependence on the tip-sample distance, z. We take the couplings with the molecular sites to decay exponentially with the height, z, as $T_{i,k,k'}(z) = T_i(z) = T_{i,0}\exp\left(-\mu z\right)$. In our simulations we fix μ_i and the $T_{i,0}$ so that $T_{\mathrm{Nc}}(z) = 3$ for all z and $T_i(z)$ has value 3 at closest distance and decays exponentially to 1 at the furthest distance (2 Å above). The current is then computed in second order as:

$$I_{inel}(V) = \sum_{\alpha,\alpha',\eta=x,y,z} P_\alpha |\langle\alpha| \sum_{i\in\mathrm{site}} T_i(z)\hat{S}_{i,\eta}|\alpha'\rangle|^2 F_{\alpha,\alpha'}(V), \tag{8}$$

where

$$F_{\alpha,\beta}(V) = \left(\frac{V - E_\alpha + E_{\alpha'}}{1 - \exp\left(-\beta(V - E_\alpha + E_{\alpha'})\right)} + \frac{V + E_\alpha - E_{\alpha'}}{1 - \exp\left(\beta(V + E_\alpha - E_{\alpha'})\right)}\right), \tag{9}$$

and P_α are the populations of the eigenstates α at thermal equilibrium, $P_\alpha = \exp\left(-\beta E_\alpha\right)$.

Sample preparation

The Au(111) crystal was prepared by several cycles of sputtering with Ar+ ions and subsequent annealing at ≈500 °C in ultra-high vacuum (UHV). The precursor molecules, 6,14-Bis(2,6-dimethylphenyl)-3,11-diphenyldibenzo[hi,st]ovalene (DBOV-Ph), were sublimed into the UHV chamber from a Knudsen cell via heating at 400 °C for 10 minutes, resulting in a coverage of ≈0.3 ML. The Au(111) surface was held at room temperature during deposition. Subsequently, the sample was annealed at ≈250 °C for 12 minutes to facilitate both the formation of planar molecules via cyclodehydrenation, and the oxidative ring closure reaction which resulted in the formation of the dimers D1, D2a and D2b (Fig. S17).

SPM Measurements

All scanning probe microscopy (SPM) measurements were performed in a SPECS-JT microscope at a measuring temperature of 4 K using a Kolibri sensor (f0 ≈ 1 MHz, Q 160k, K ≈1800 N/m, 50 pm amplitude modulation). STS, dI/dV maps, and ncAFM measurements were all performed by first functionalizing the tip with a carbon-monoxide (CO) molecule. A lock-in method was used for both STS and dI/dV maps using Vmod = 1 mV, fmod = 723 Hz.

NiCp2 Measurements

Nickelocene (NiCp2) molecules were deposited from a tantalum pocket kept at room temperature directly onto the sample in the microscope (T¡10 K). The tip was functionalized with a NiCp2 molecule by repeatedly scanning over an individual molecule on the Au(111) surface with Vb ¡ 4 mV, It ¡ 50 pA until spontaneous functionalization occurred. NiCp2 tips were characterised by the sharpness of their imaging, by their stability in ncAFM signal as measured by forward and backward $\Delta f(\Delta z)$ curves, and finally by the presence of inelastic

signatures at ±4 mV on bare Au(111) (see Fig. S18 in the SOM). A lock-in method was used to record d2I/dV2 for acquiring both spectra (e.g. Figure 1) and maps (e.g. Figure 2g), with Vmod = 1 mV, fmod = 723 Hz.

2 Simulations

We have performed multireference CASCI+NEVPT2 calculations to describe electronic structure of molecules **D1**, **D2a** and **D2b**.

Ab initio calculations for Dimer D1

The CASCI(12,12) calculations predict dimer D1 to be a diradical with a singlet ground state and a triplet excited state at 12 meV (see Fig. 2 in the main text). We employed the active space of 12 frontiers KS orbitals obtained from spin unpolarized DFT calculation. Fig. S1 shows six of twelve frontier KS orbitals employed in the CASCI calculation.

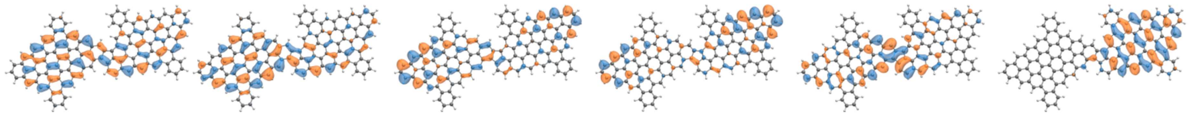

Figure S1: Six frontier orbitals of the active space for the CASCI calculation

The occupancy of natural Orbitals (see Fig. S2) predicts diradical character for the singlet ground state.

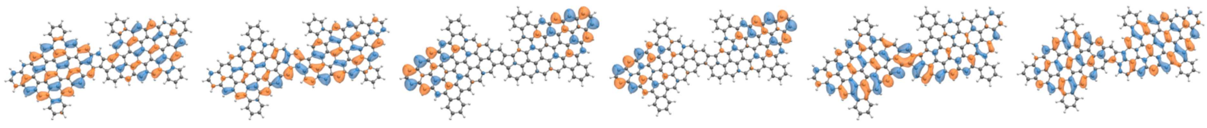

Figure S2: Natural Orbitals and their occupations, showing the radicality of the molecule

Fig. S3 represents calculated Natural Transition Orbitals (NTOs) corresponding to the spin excitation from the singlet ground state to the first excited triplet state. From two

dominant NTOs, we simulate the dI/dV map with the Probe Particle code described in the Methods section of the main text.

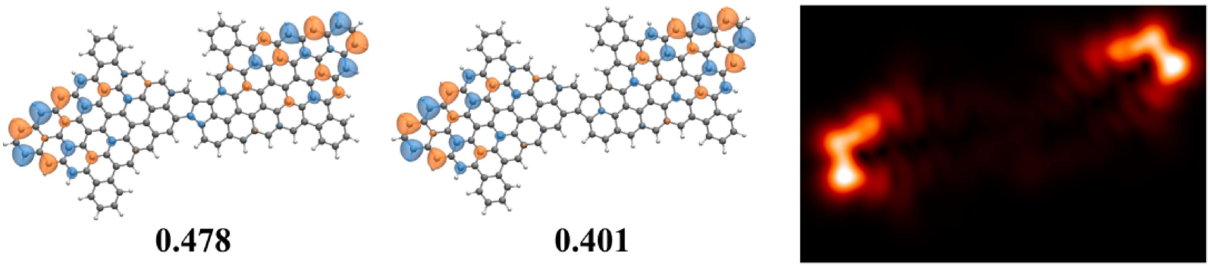

Figure S3: Dominant NTOs and assoaciated dI/dV map for the singlet-triplet transition.

Ab initio calculations for Dimers D2a, D2b

The ab initio CASCI(11,11) calculations predict both dimers D2a and D2b to have a doublet ground state and quartet first excited state at 4 meV.In Fig. S4 and Fig. S5 we show five frontier orbitals, out of a total of 11, obtained from a DFT spin-restricted calculation for D2a and D2b, respectively.

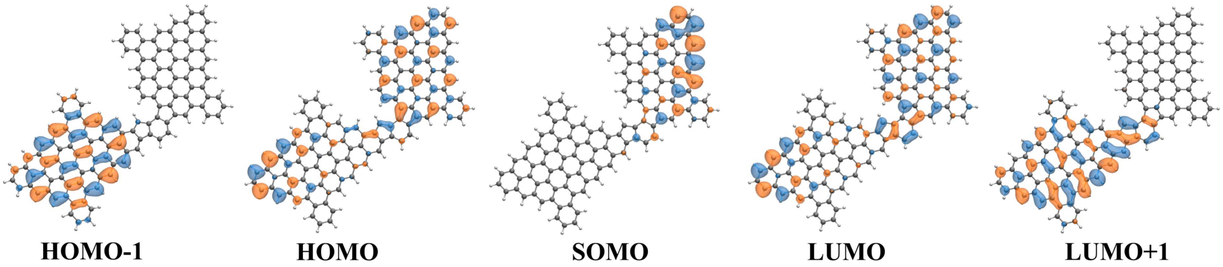

Figure S4: Five frontier DFT orbitals for **D2a** employed as the active space for the CASCI calculation

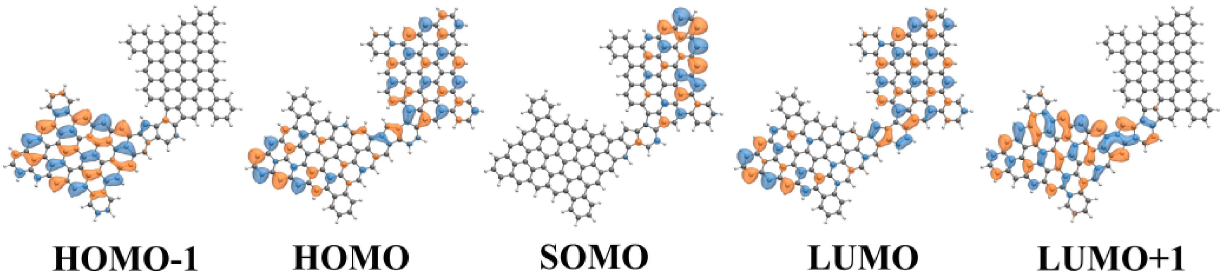

Figure S5: Five frontier DFT orbitals for **D2b** employed as the active space for the CASCI calculation

The corresponding Natural Orbitals, revealing the triradical nature of the doublet ground state, are shown respectively on Fig. S6 and Fig. S7

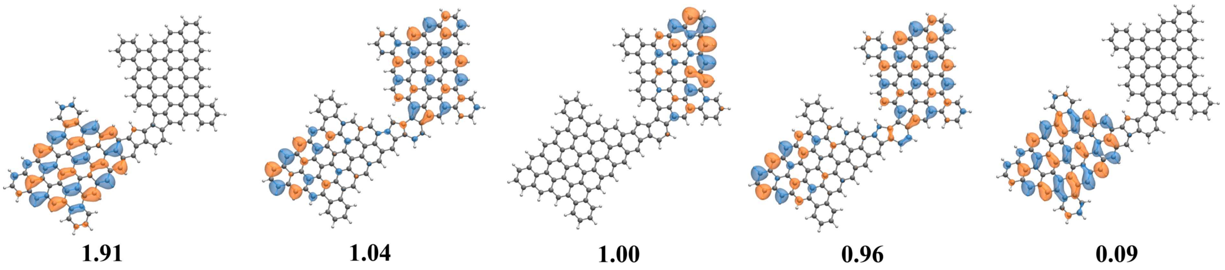

Figure S6: Natural orbitals for **D2a**

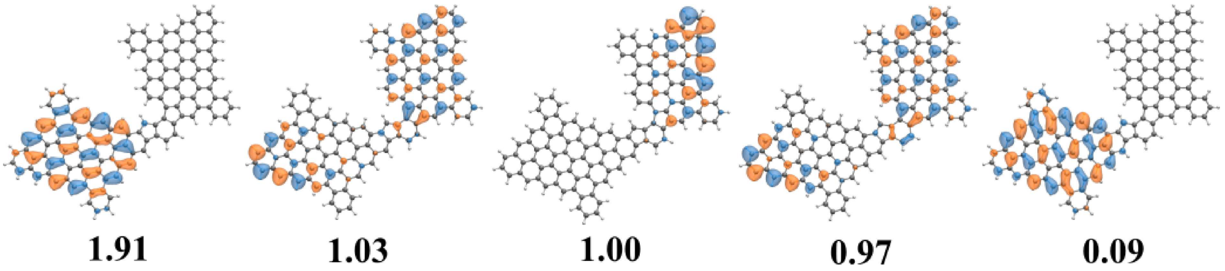

Figure S7: Natural Orbitals for **D2b**

From the CASCI wavefunctions for the doublet and quartet state, we construct the NTOs associated to the spin transition from the ground state to the first excited state as well as the corresponding simulated dI/dV map.

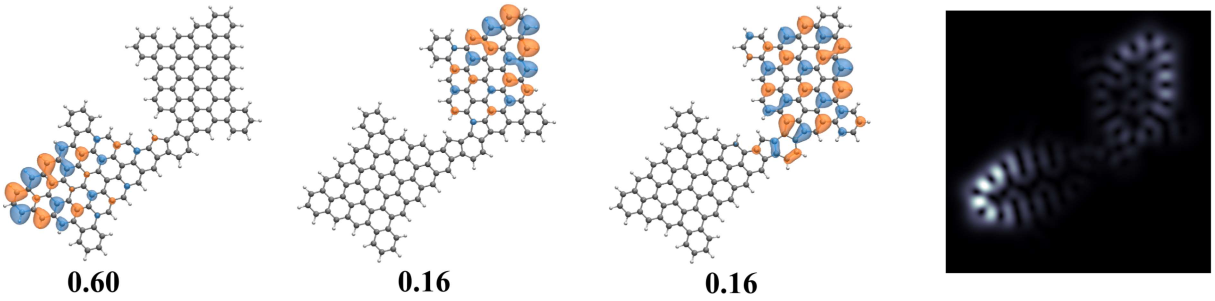

Figure S8: Dominant NTOs and associated simulated dI/dV map for the spin excitation for **D2a**

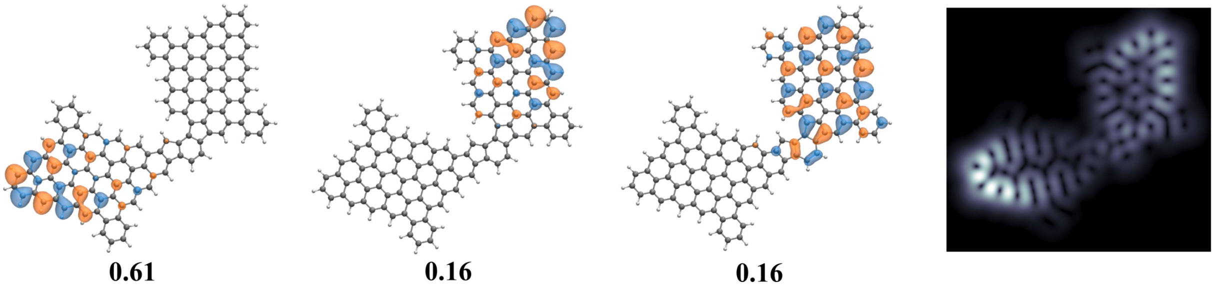

Figure S9: Dominant NTOs and associated simulated dI/dV for the spin excitation for **D2b**

Next, we turn our attention to the Kondo signals for the doublet and quartet state. In Figs. S10, Fig. S11, Fig. S12 and S13 we show Kondo orbitals and their corresponding dI/dV maps for the doublet and quartet state of dimers **D2a** and **D2b**.

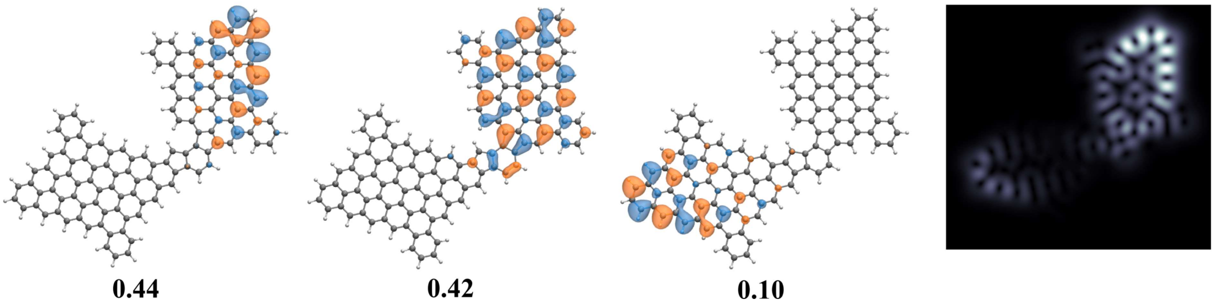

Figure S10: Kondo orbitals and associated dI/dV map for the **doublet** state in Dimer **D2b**

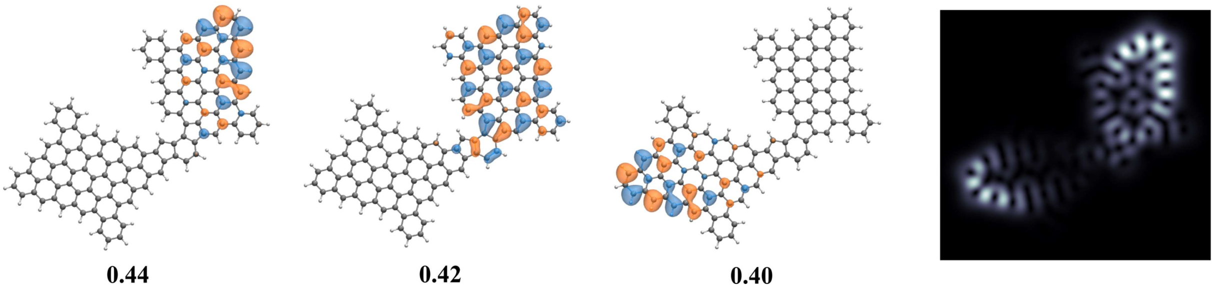

Figure S11: Kondo orbitals and associated dI/dV map for the **quartet** state in Dimer **D2b**

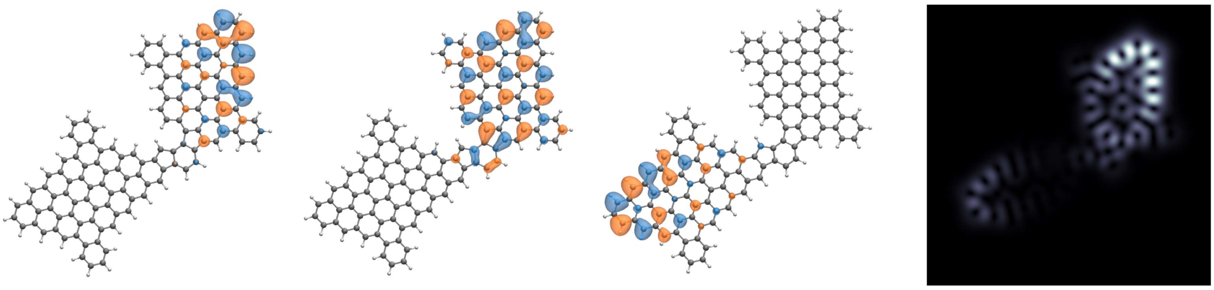

0.44 **0.42** **0.10**

Figure S12: Kondo orbitals and associated dI/dV map for the **doublet** state in Dimer **D2a**

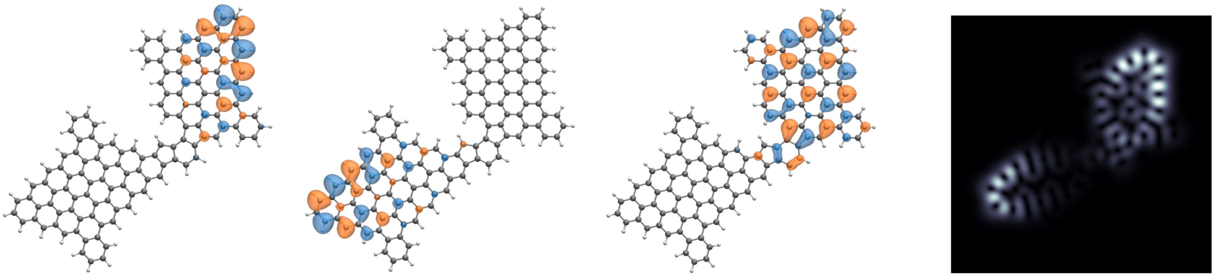

0.44 **0.42** **0.40**

Figure S13: Kondo orbitals and associated dI/dV map for the **quartet** state in Dimer **D2a**

Importantly, it is evident that the spatial distribution of the spin excitation and Kondo signal for the doublet and quartet state cannot help to discriminate from experimental measurements the ground state. This motivates the study of the spin models and the interaction with the NiCp2 as discussed in the next section.

Spin model for dimer D1

The renormalization of the NiCp2 excitation lines, shown in Fig. 2e,f, characterizes the dimer **D1** as a dimer with an anti-ferromagnetic interaction and a local radical. Indeed, a diradical with identical excitation but with ferromagnetic J (i.e, a triplet ground state) presents a very different spectra, as shown in Fig. S14.

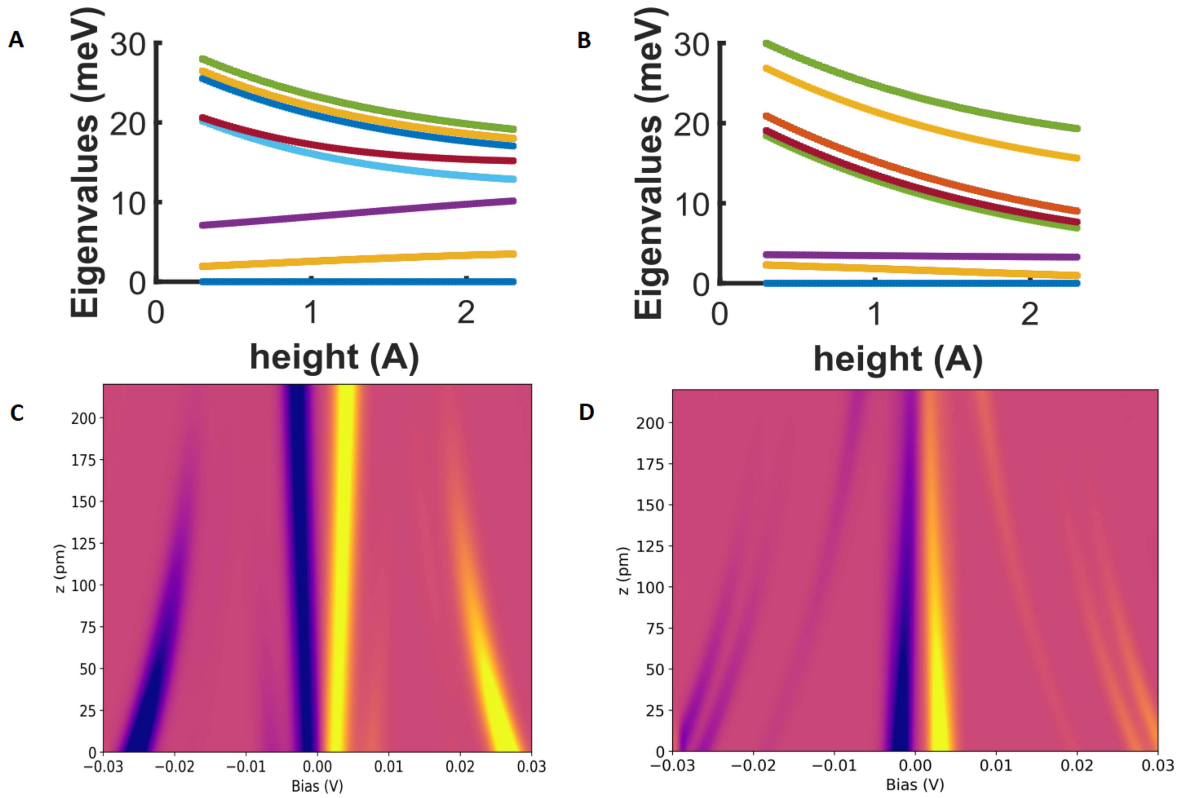

Figure S14: a),b) Eigenvalues of the Heisenberg model for the diradical-NiCp2 interaction for an anti-ferromagnetic and ferromagnetic diradical, respectively. c),d) simulated d^2I/dV^2 for the antiferromagnetic and ferromagnetic diradicals, respectively.

As mentioned in the main text, the renormalization of the excitation lines of the $NiCp_2$ indicates also that exchange interaction is local. It is instructive to discuss the spin model for the dimer introduced in the main text, $\hat{H}_1(\Delta z)$, considering two interaction models including the local interaction to individual spin of molecule $\hat{H}_{\text{int,loc}} = J(\Delta z)\vec{\hat{S}}_{\text{Nc}} \cdot \vec{\hat{S}}_1$ and interaction including both molecular spins $\hat{H}_{\text{int,global}} = J(\Delta z)\vec{\hat{S}}_{\text{Nc}} \cdot \left(\vec{\hat{S}}_1 + \vec{\hat{S}}_2\right)$. As shown in Fig. S15 a) (local interaction) and b) (global interaction), only a local exchange coupling results in the renormalization of the spin excitation of NiCp2 tip along the tip approach. The reason lies in the fact that the the total spin of the molecule, S^2_{mol}, is only a good quantum number for any value of J for the case of the global interaction. Indeed, the locally interactive model breaks this symmetry. To demonstrate this in details, we show in Fig. S15 c),d) the projection of the first excited state into the pure spin states. This

projections are calculated for each Δz as follows: Let us call Ψ_1 to the first excited state of the system $NiCp_2$+Dimer for a generic value of J. The pure spin manifolds of states, $|S_{\mathrm{z,Nc}} = 0, S^2_{\mathrm{M}} = 0\rangle, |S_{\mathrm{z,Nc}} = \pm 1, S^2_{\mathrm{M}} = 0\rangle, |S_{\mathrm{z,Nc}} = 0, S^2_{\mathrm{M}} = 2\rangle, |S_{\mathrm{z,Nc}} = \pm 1, S^2_{\mathrm{M}} = 2\rangle$ are obviously eigenstates of the system for $J = 0$. We can then calculate the projections:

$$P_{0,0} = \langle S_{z,\mathrm{Nc}} = 0, S^2_{\mathrm{M}} = 0 | \Psi_1 \rangle \tag{10}$$

$$P_{1,0} = \langle S_{z,\mathrm{Nc}} = \pm 1, S^2_{\mathrm{M}} = 0 | \Psi_1 \rangle \tag{11}$$

$$P_{0,1} = \langle S_{z,\mathrm{Nc}} = 0, S^2_{\mathrm{M}} = 2 | \Psi_1 \rangle \tag{12}$$

$$P_{1,1} = \langle S_{z,\mathrm{Nc}} = \pm 1, S^2_{\mathrm{M}} = 2 | \Psi_1 \rangle . \tag{13}$$

For $J = 0$, we have $P_{1,0} = 1$ and 0 for the rest of projectors. As J increases, for the case of a global interaction we find that the projectors remain constant, since S^2_{mol} is a symmetry of the system (see Fig. S15d)). However, for the local interaction, these projectors depend on J, causing the mixing of pure spin states responsible for the renormalization of the excitation lines.

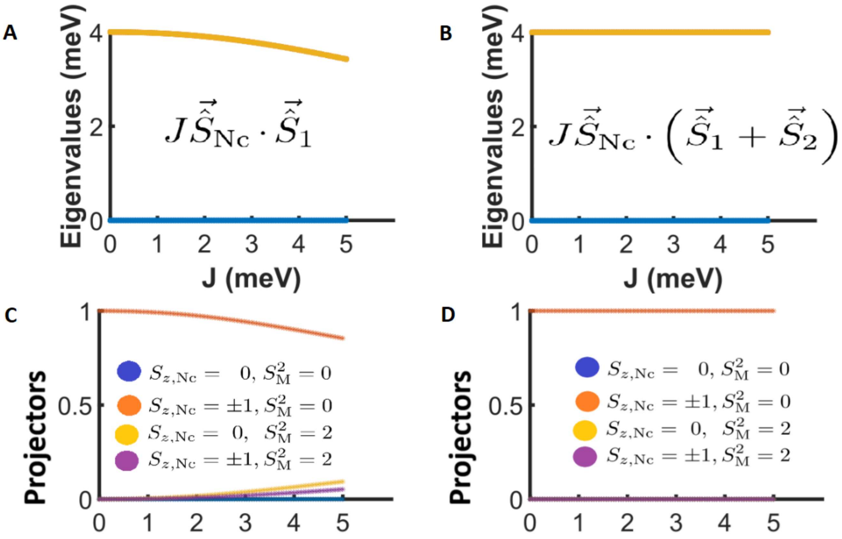

Figure S15: a) and b): First three eigenvalues for the Heisenberg model of the interaction of a $NiCp_2$ with an antiferromagnetic dimer. In a), the S=1 of the $NiCp_2$ couples to only one of the spins, whereas in b) the S=1 couples to the total spin of the dimer. c,d) Projection of the first excited state of the system over the pure spin states for the case of local and global coupling, respectively. We show in blue, orange, yellow and purple, respectively, the projectors $P_{0,0}, P_{1,0}, P_{0,1}, P_{1,1}$ (see text).

Mapping dimers D2 to a spin model

We can construct a basis of maximally localized orbitals to fit the CASCI energy spectra to the spin models. The localization is only relevant for the first three frontier orbitals labeled as HOMO, SOMO, LUMO in Figs. S4,S5. We construct linear combinations of three HOMO, SOMO, LUMO orbitals yielding orthonormal orbitals with maximal localization. The resulting frontier orbitals are shown respectively on Fig. S16 and Fig. S17.

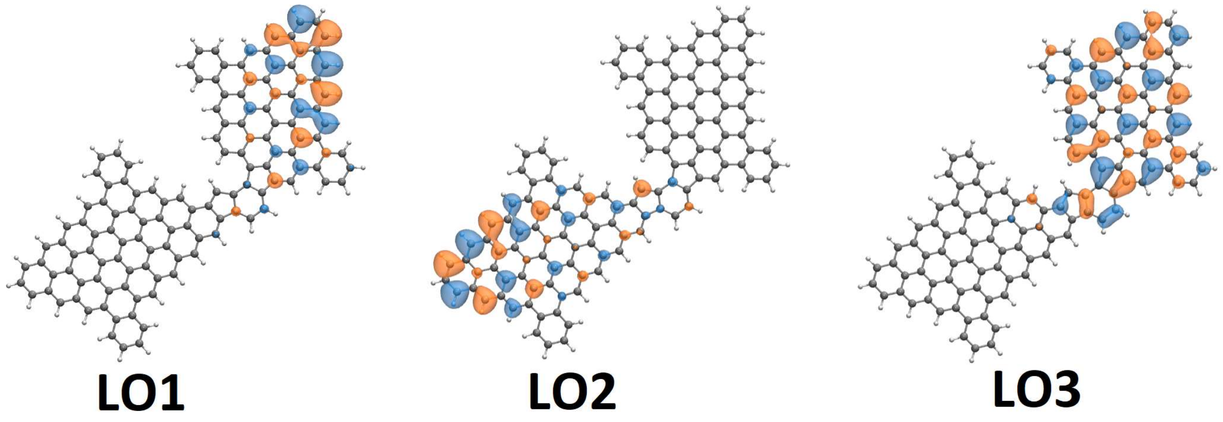

Figure S16: Maximally localized molecular orbitals obtained from the rotation of three frontiers KS orbitals for **D2a**.

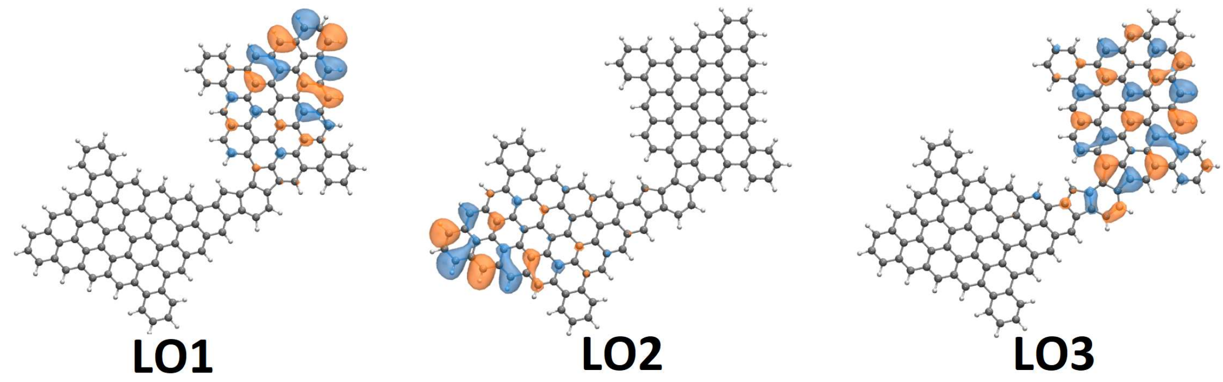

Figure S17: Maximally localized molecular orbitals obtained from the rotation of three frontiers KS orbitals for **D2b**.

From the CASCI calculations, we can compute the spin correlations in the basis of the maximally localized orbitals (LO) for both the duplet and quartet state (see Tables 1 and 2, respectively):

$$\langle \hat{\vec{S}}_i \cdot \hat{\vec{S}}_j \rangle - \langle \hat{\vec{S}}_i \rangle \cdot \langle \hat{\vec{S}}_j \rangle$$

, where i, j label the localized orbitals LO1,LO2 and LO3. Note that the spin models reproduce this information, as well as the spectral gap.

Robustness of spectroscopic features for Dimers D2a, D2b

The spectroscopic features shown in Fig. 3 of the main text do not depend strongly on

Table S1: Calculated spin correlations between maximally localized molecular orbitals for the quartet state obtained from the CASCI calculation.

$\mathbf{DOUBLET}white$	**LO1**	**LO2**	**LO3**
**LO1**	$green!200.64$	$blue!20-0.44$	$green!200.14$
**LO2**	$blue!20-0.44$	$green!200.72$	$blue!20-0.44$
**LO3**	$green!200.14$	$blue!20-0.44$	$green!200.64$

Table S2: Calculated spin correlations between maximally localized molecular orbitals for the quartet state obtained from the CASCI calculation.

$\mathbf{QUARTET}white$	**LO1**	**LO2**	**LO3**
**LO1**	$green!200.72$	$green!200.22$	$green!200.22$
**LO2**	$green!200.22$	$green!200.72$	$green!200.22$
**LO3**	$green!200.14$	$green!200.22$	$green!200.672$

the value of the spin excitation. This makes the identification of the ground state reliable. In Fig. S18, we show simulated d^2I/dV^2 maps, akin to the ones in Fig.3 of the main text, for a range of values of the molecular spin excitation. All these maps are constructed by applying co-tunneling theory for a range of values of z to the spin models modeling the molecule-NiCp2 interaction.

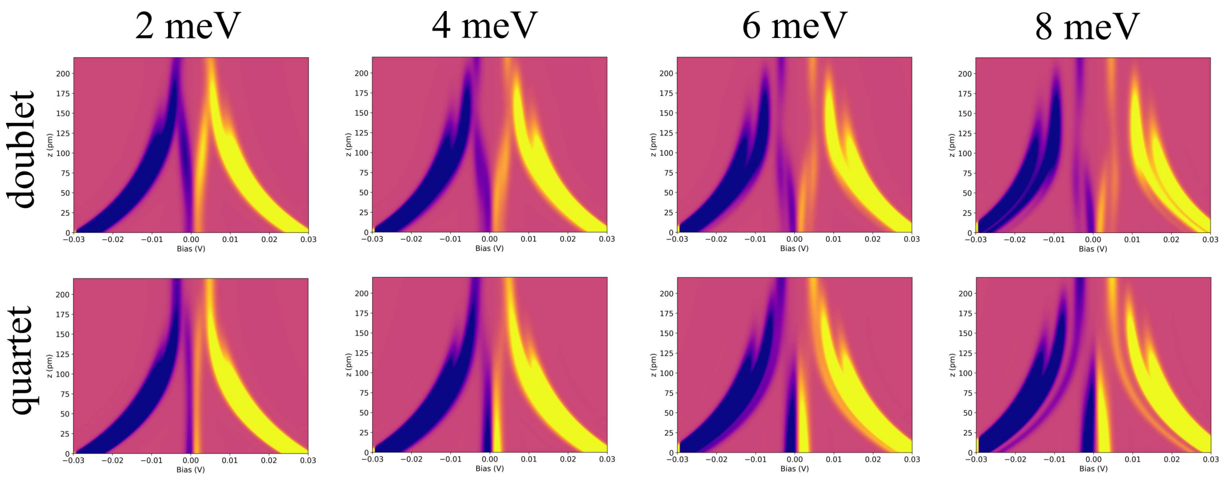

Figure S18: Simulated d^2I/dV^2 for the interaction of NiCp2 tip with the triradicals doublet (upper row) and quartet (bottom row), described by the model spin hamiltonians. Each column corresponds to a different spin excitation in the molecule.

Likewise, such features are also robust under perturbations of the exchange interaction of the radicals with the NiCp2 tip; i.e, changing the decay constant λ in the exponential law, $J_{\mathrm{Nc},i}(\Delta z) = J_{0,1} \exp\left(-\lambda \Delta z\right)$ does not affect the qualitative results, as shown in Fig. S19.

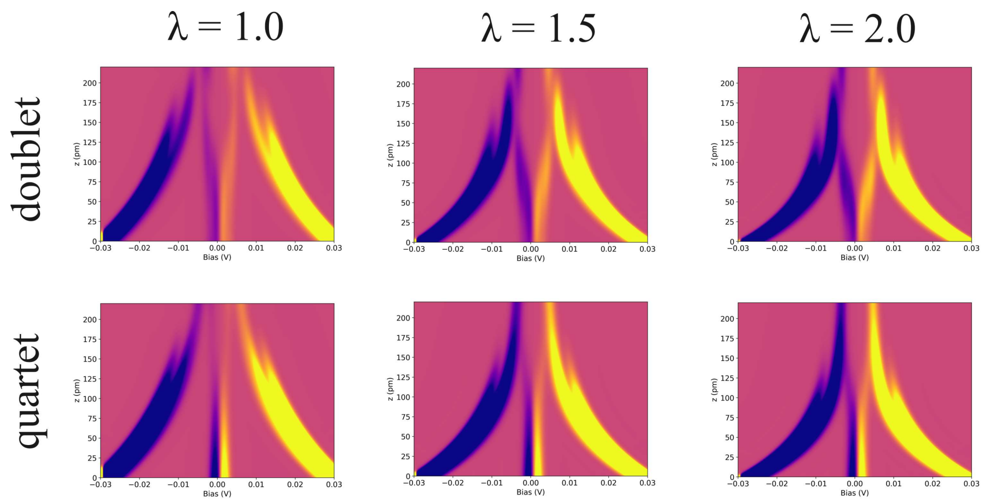

Figure S19: Simulated d^2I/dV^2 for the interaction of NiCp2 tip with the triradicals doublet (upper row) and quartet (bottom row), described by the model spin hamiltonians. Each column corresponds to a different decay rates for the exponentially decreasing excange interaction with respect to the height.

3 Experimental details

On-surface synthesis of D1, D2a and D2b

The on-surface synthesis of the dimers **D1**, **D2a**, and **D2b** are shown schematically in Fig. S20. The precursor molecules, 6,14-Bis(2,6-dimethylphenyl)-3,11-diphenyldibenzo[hi,st]ovalene (DBOV-Ph), were deposited on the Au(111) surface at room temperature. Subsequent annealing of the sample at 250°C facilitated cyclodehydrogenation processes which resulted in planar aromatic molecules. This temperature also allowed for oxidative ring closure reactions between pairs of molecules (and methyl cleavage in the case of **D2** dimers) to form the dimers investigated in this study.

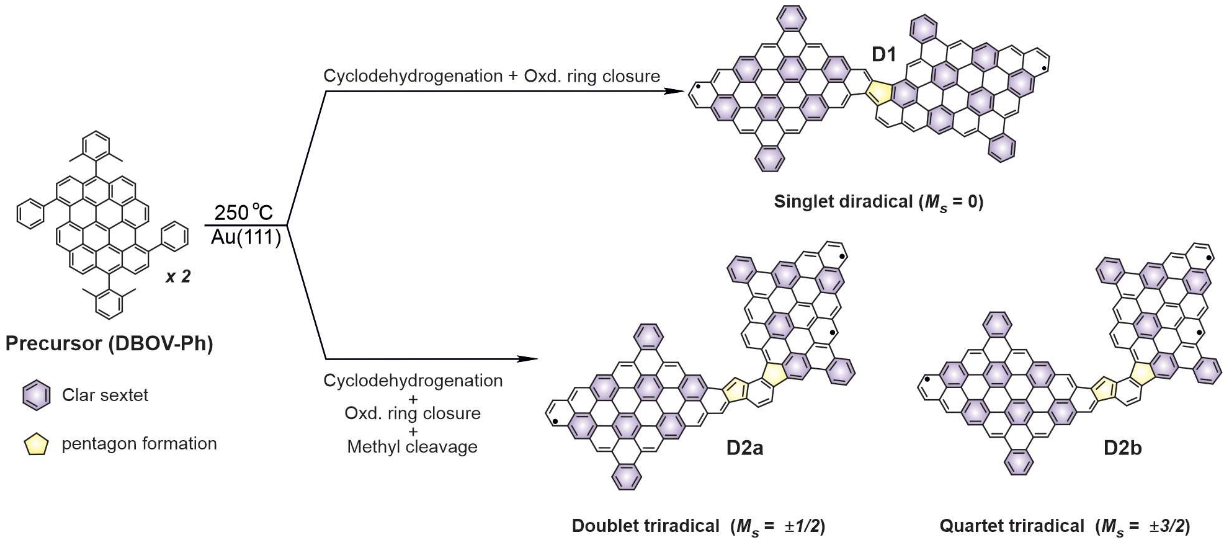

Figure S20: Scheme for the on-surface synthesis of the nanographene dimers **D1**, **D2a**, and **D2b** using the precursor molecule **DBOV-Ph**.

Experimental dI/dV maps of D2 dimers

Experimental constant-height dI/dV maps of the two **D2** dimers are shown in Figure S21. These can be compared directly with the Kondo orbitals and NTOs shown for each **D2** dimer shown in Section 1. While these experimental maps show good agreement with the theoretical maps, the similarity between the maps calculated using the doublet state and the maps calculated quartet state are too similar to use these spatial distributions as a means of conclusively determining the ground state of each molecule.

Reference $NiCp_2$ Measurements

All measurements shown in the main text were performed with the same $NiCp_2$ tip. Prior to performing measurements, the $NiCp_2$ tip was first characterized upon bare Au(111), and at a region of dimer **D2a** without spin-density as shown in Figure S22.

On Au(111), $\Delta f(\Delta z)$ curves confirm the stability of the $NiCp_2$ with smooth curves free from hysteresis between forward and backward sweeps produced (Figure S22b). Tip-height dependent d^2I/dV^2 measurements on bare Au(111) confirmed no magnetic interaction between tip and sample with the typical $NiCp_2$ inelastic signatures at ±4 mV observed for all tip-sample distances (Figure S22c).

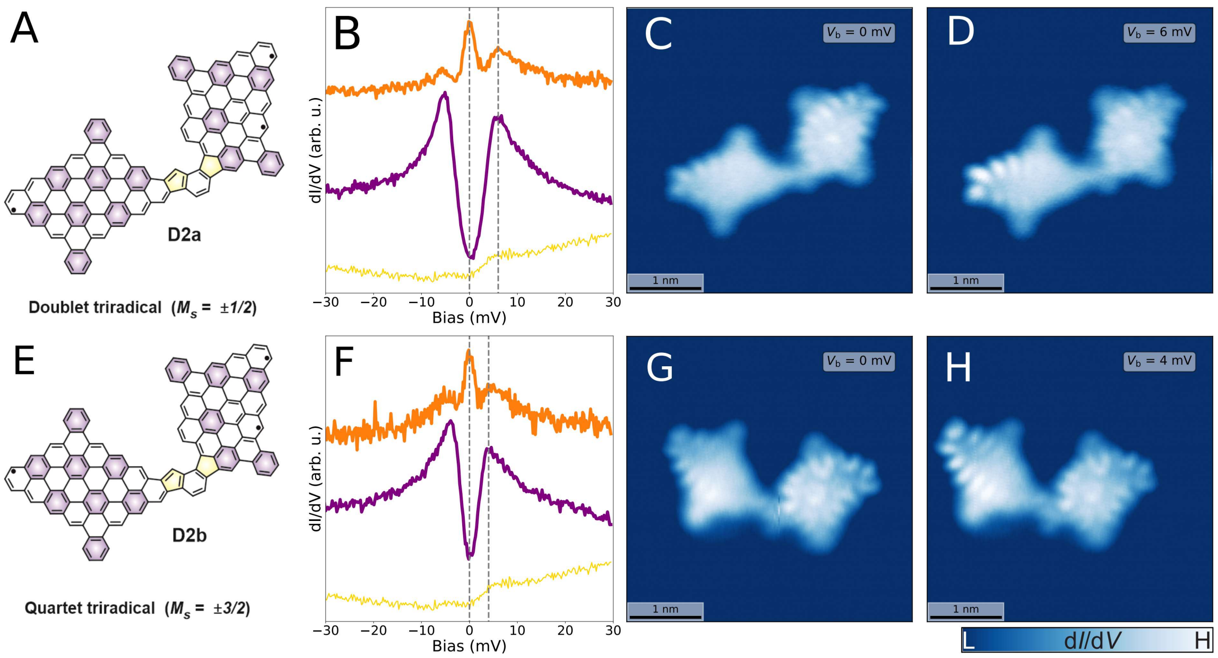

Figure S21: Experimental dI/dV maps corresponding to Kondo and inelastic excitations of **D2a** and **D2b**. **a,e** Chemical sketch of **D2a** and **D2b** dimers, respectively. **b,f** STS measurements of **D2a** and **D2b** dimers, respectively (as shown in main text Fig. 3). Grey dashed lines indicate biases at which dI/dV maps were acquired. **c,d,g,h** Constant-height dI/dV maps acquired with a CO-tip corresponding to Kondo and inelastic spin excitation features for **D2a** (c,d) and **D2b** (g,h). $V_{\mathrm{mod}} = 1$ mV.

Further characterisation of the $NiCp_2$ tip was performed at a region of the **D2a** dimer with no spin density. Here, equivalent tip-molecule distances could be obtained as were used for the measurements shown in the main text. The smooth appearance and absence of hysteresis between forward and backward sweeps in the $\Delta f(\Delta z)$ spectrum in Figure S22d confirms the stability of the $NiCp_2$ molecule at these tip-sample distances. Similar curves were acquired before each tip-height dependent map shown in the main text (Figures 1, 2e, 3e, and 3k). At this site of the **D2a** dimer, there is no magnetic interaction between the tip and sample as seen from the tip-height dependent $\mathrm{d}^2I/\mathrm{d}V^2$ measurements in Figure S22e (in contrast to the measurements shown in the main text). This is further evidence of the ability of $NiCp_2$-functionalized tips to act as magnetic sensors with unprecedented spatial resolution.

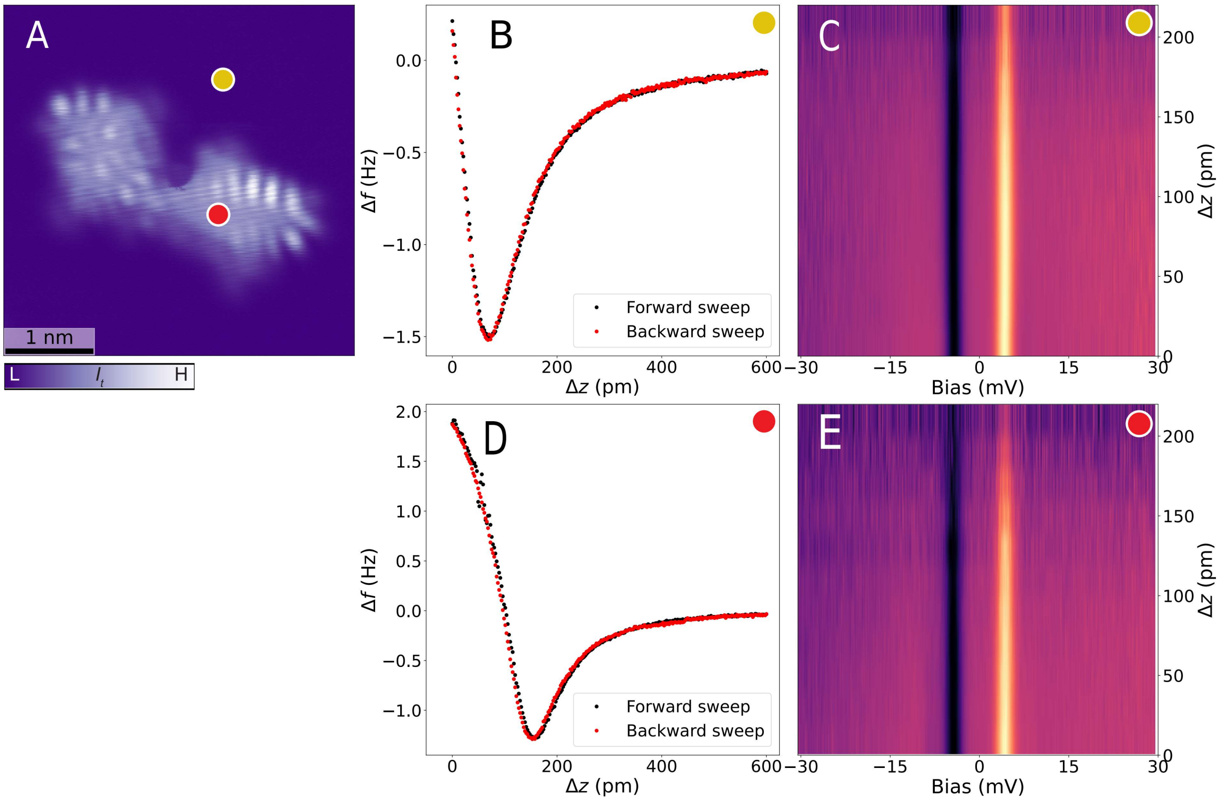

Figure S22: Reference $NiCp_2$ measurements. **a** Constant-height STM image of dimer **D2a** acquired with a $NiCp_2$-functionalized tip (V_b = 20 mV). **b** $\Delta f(\Delta z)$ measurement acquired on bare Au(111) (gold marker in a). **c** Tip height-dependent map composed of a series of eleven $NiCp_2$ d^2I/dV^2 spectra acquired on bare Au(111) (gold marker in a). **d** $\Delta f(\Delta z)$ measurement acquired on part of the **D2a** dimer with no spin density (red marker in a). **e** Tip height-dependent map composed of a series of eleven $NiCp_2$ d^2I/dV^2 spectra acquired on part of the **D2a** dimer with no spin density (red marker in a). Δz ranges in c,e correspond directly to Δz ranges shown in b,d respectively.

Figure S23 shows the raw spectra used to compose the maps shown in main text Fig. 3e and k.

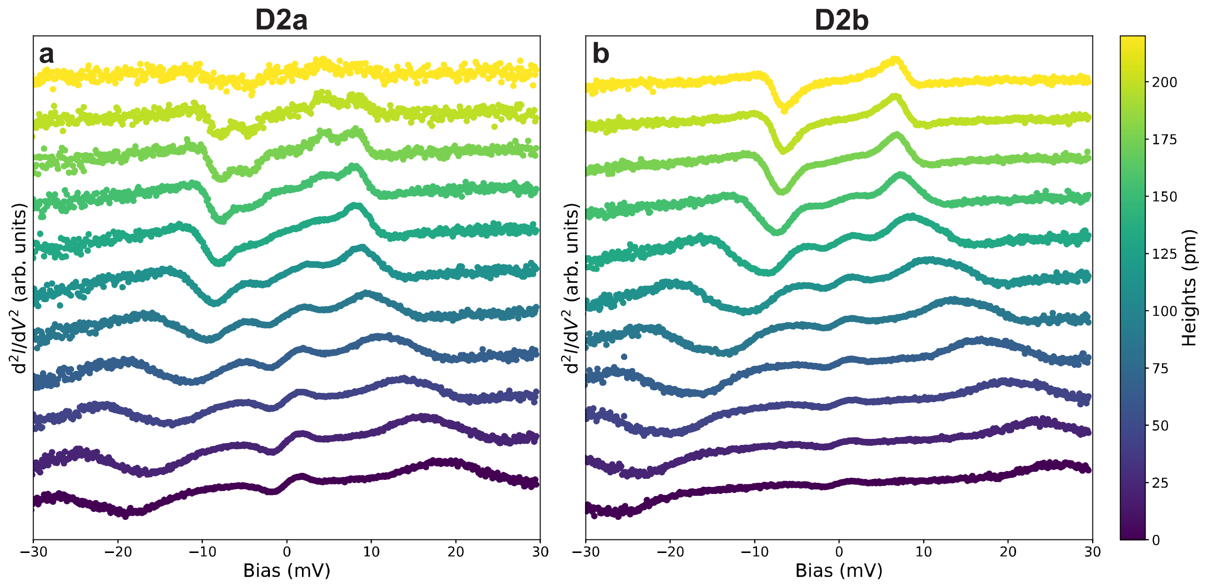

Figure S23: Tip-height dependent d^2I/dV^2 spectra acquired with a $NiCp_2$ terminated tip. a,b These measurements show the tip-height dependent $NiCp_2$ STS measurements performed on molecules D2a and D2b respectively. The color of the curves correspond to the tip-sample distance (purple smallest tip-sample distance, yellow largest tip-sample distance).

References

(S1) Blum, V.; Gehrke, R.; Hanke, F.; Havu, P.; Havu, V.; Ren, X.; Reuter, K.; Scheffler, M. Ab initio molecular simulations with numeric atom-centered orbitals. *Computer Physics Communications* **2009**, *180*, 2175–2196.

(S2) Perdew, J. P.; Ernzerhof, M.; Burke, K. Rationale for mixing exact exchange with density functional approximations. *The Journal of Chemical Physics* **1996**, *105*, 9982–9985.

(S3) Kumar, M.; Soler-Polo, D.; Lozano, M.; Monino, E.; Veis, L.; Jelinek, P. Multireference Theory of Scanning Tunneling Spectroscopy Beyond One-Electron Molecular Orbitals: Can We Image Molecular Orbitals? *Journal of the American Chemical Society* **2025**, *147*, 24993–25003.

(S4) Neese, F. The ORCA program system. *WIREs Computational Molecular Science* **2012**, *2*, 73–78.

(S5) Angeli, C.; Cimiraglia, R.; Evangelisti, S.; Leininger, T.; Malrieu, J.-P. Introduction of n-electron valence states for multireference perturbation theory. *The Journal of Chemical Physics* **2001**, *114*, 10252–10264.

(S6) Löwdin, P.-O. Quantum Theory of Many-Particle Systems. I. Physical Interpretations by Means of Density Matrices, Natural Spin-Orbitals, and Convergence Problems in the Method of Configurational Interaction. *Phys. Rev.* **1955**, *97*, 1474–1489.

(S7) Martin, R. L. Natural transition orbitals. *The Journal of Chemical Physics* **2003**, *118*, 4775–4777.

(S8) Calvo-Fernández, A.; Kumar, M.; Soler-Polo, D.; Eiguren, A.; Blanco-Rey, M.; Jelínek, P. Theoretical model for multiorbital Kondo screening in strongly correlated molecules with several unpaired electrons. *Phys. Rev. B* **2024**, *110*, 165113.

(S9) Krejčí, O.; Hapala, P.; Ondráček, M.; Jelínek, P. Principles and simulations of high-resolution STM imaging with a flexible tip apex. *Physical Review B* **2017**, *95*.

(S10) Fernández-Rossier, J. Theory of Single-Spin Inelastic Tunneling Spectroscopy. *Phys. Rev. Lett.* **2009**, *102*, 256802.

(S11) Pinar Solé, A.; Kumar, M.; Soler-Polo, D.; Stetsovych, O.; Jelínek, P. Nickelocene SPM tip as a molecular spin sensor. *Journal of Physics: Condensed Matter* **2024**, *37*, 095802.

(S12) Appelbaum, J. A. Exchange Model of Zero-Bias Tunneling Anomalies. *Phys. Rev.* **1967**, *154*, 633–643.
